# Non-viral Gene Therapy for Osteoarthritis

**DOI:** 10.3389/fbioe.2020.618399

**Published:** 2021-01-13

**Authors:** Ilona Uzieliene, Ursule Kalvaityte, Eiva Bernotiene, Ali Mobasheri

**Affiliations:** ^1^Department of Regenerative Medicine, State Research Institute Centre for Innovative Medicine, Vilnius, Lithuania; ^2^Research Unit of Medical Imaging, Physics and Technology, Faculty of Medicine, University of Oulu, Oulu, Finland; ^3^Departments of Orthopedics, Rheumatology and Clinical Immunology, University Medical Center Utrecht, Utrecht, Netherlands; ^4^Centre for Sport, Exercise and Osteoarthritis Versus Arthritis, Queen's Medical Centre, Nottingham, United Kingdom

**Keywords:** osteoarthritis, cartilage, chondrocyte, non-viral, gene therapy

## Abstract

Strategies for delivering nucleic acids into damaged and diseased tissues have been divided into two major areas: viral and non-viral gene therapy. In this mini-review article we discuss the application of gene therapy for the treatment of osteoarthritis (OA), one of the most common forms of arthritis. We focus primarily on non-viral gene therapy and cell therapy. We briefly discuss the advantages and disadvantages of viral and non-viral gene therapy and review the nucleic acid transfer systems that have been used for gene delivery into articular chondrocytes in cartilage from the synovial joint. Although viral gene delivery has been more popular due to its reported efficiency, significant effort has gone into enhancing the transfection efficiency of non-viral delivery, making non-viral approaches promising tools for further application in basic, translational and clinical studies on OA. Non-viral gene delivery technologies have the potential to transform the future development of disease-modifying therapeutics for OA and related osteoarticular disorders. However, further research is needed to optimize transfection efficiency, longevity and duration of gene expression.

## Introduction

Osteoarthritis (OA) is a leading cause of disability across the world and a major cost contributor to health and social care systems (Hunter and Bierma-Zeinstra, 2019). In terms of prevalence OA is the most common chronic and degenerative disease of synovial joints (Bijlsma et al., 2011). The incidence of OA is rising because of the aging population and the epidemic of obesity (Messier, 2008; King et al., 2013; Bliddal et al., 2014). Degradation and loss of articular cartilage is a hallmark of OA, resulting in severe pain, loss of joint function and impaired quality of life (Buckwalter et al., 2005).

There are a variety of pharmacological and non-pharmacological treatments for OA (Rannou and Poiraudeau, 2010; Mobasheri, 2013b; Ghouri and Conaghan, 2019b). Official recommendations for OA treatment are often divided into non-pharmacological, pharmacological, and surgical interventions (Bijlsma et al., 2011; Buttgereit et al., 2015). Among the available updated guidelines for the management of knee OA, those from OARSI (Bannuru et al., 2019) and ESCEO (Bruyère et al., 2019) were updated in 2019 and the ACR guidelines were updated in 2020 (Kolasinski et al., 2020). In the absence of disease-modifying OA drugs (DMOADs), most clinical guidelines recommend the provision of education, physical therapy and weight management in their core treatment recommendations (Nelson et al., 2014). Since there are no effective pharmacological treatments for OA, significant effort has gone into the development of biological drugs (Mobasheri, 2013a) and cell-based therapies (Salem et al., 2019; Grässel and Muschter, 2020).

This concise mini review summarizes the most significant papers on advances of a non-viral gene delivery studies in OA. Advantages and disadvantages of different types of gene modifications for the treatment of OA are summarized and the future perspectives and directions of this rapidly developing field are reviewed.

## Current Therapeutic Approaches in OA

Therapeutic approaches in OA are aimed at addressing symptoms and improving structural features of the diseased joint. Surgical correction and realignment of varus and valgus malalignment in the joint and other surgical procedures such as microfracture, autologous osteochondral grafting (mosaicplasty), using xenografts, or biomaterial implants as well as partial or total joint replacement surgery are the most effective and current surgical interventions to improve quality of life in OA patients (Oztürk et al., 2006; Erggelet and Vavken, 2016; Grässel and Muschter, 2020).

For the majority of OA patients, the only pharmacological options include painkillers such as acetaminophen and non-steroidal anti-inflammatory drugs (NSAIDs) (McAlindon et al., 2014). However, these drugs are not effective for disease modification and the long-term use of acetaminophen and NSAIDs for osteoarthritis is associated with adverse side-effects on the cardiovascular, gastrointestinal and renal systems (McCrae et al., 2018; Ghouri and Conaghan, 2019a). Furthermore, acetaminophen is weakly recommended by ESCEO (Bruyère et al., 2019) and not recommended in the most recent OARSI (Bannuru et al., 2019) and ACR (Kolasinski et al., 2020) treatment guidelines. These issues highlight the acute need for the development of newer and safer treatments for OA, placing greater emphasis on the necessity for understanding disease phenotypes, their underlying molecular endotypes and targeting the molecules and pathways associated with them (Mobasheri et al., 2019a,b; Van Spil et al., 2019).

Therapeutic agents for treating OA have traditionally been developed with different routes of administration, including oral administration, direct injection into the joint or subcutaneous injection. Indeed, there is consensus that most of the new OA therapies are designed for intra-articular injection, even though some are still used as subcutaneous injection (Paoloni et al., 2015; Yu and Hunter, 2016; Migliore et al., 2020; Zhang et al., 2020). Future gene therapy for OA is also likely to focus on the intra-articular route of delivery.

## Cell and Gene Therapy for OA

A range of gene transfer approaches are proposed as an alternative method for a targeted and sustained delivery of therapeutic agents, growth factor genes and small regulatory components as microRNAs (Grol and Lee, 2018). Methods for gene delivery include the use of viral and non-viral gene transfer systems, where viral methods are considered to be more efficient in a sustained and targeted approach for transferring a gene of interest. Viral vectors commonly include adenoviruses, herpes simplex viruses, retroviruses, and lentiviruses for transferring genes into damaged or diseased tissues. These vectors have attracted more attention due to their evolutionarily conserved and optimized machinery for targeted delivery of genes into mammalian cells. However, due to ongoing safety concerns about the use of viral vectors *in vivo*, the development of non-viral therapies is gaining more support. The delivery systems for non-viral therapies include lipid-based systems, as well as other DNA-conjugates, which are easy to handle, safe for *in vivo* studies and generally cost-effective (Raisin et al., 2016). However, these systems have been demonstrated as less effective in gene delivery (Cucchiarini et al., 2015; Grol and Lee, 2018).

**Figure 1:**
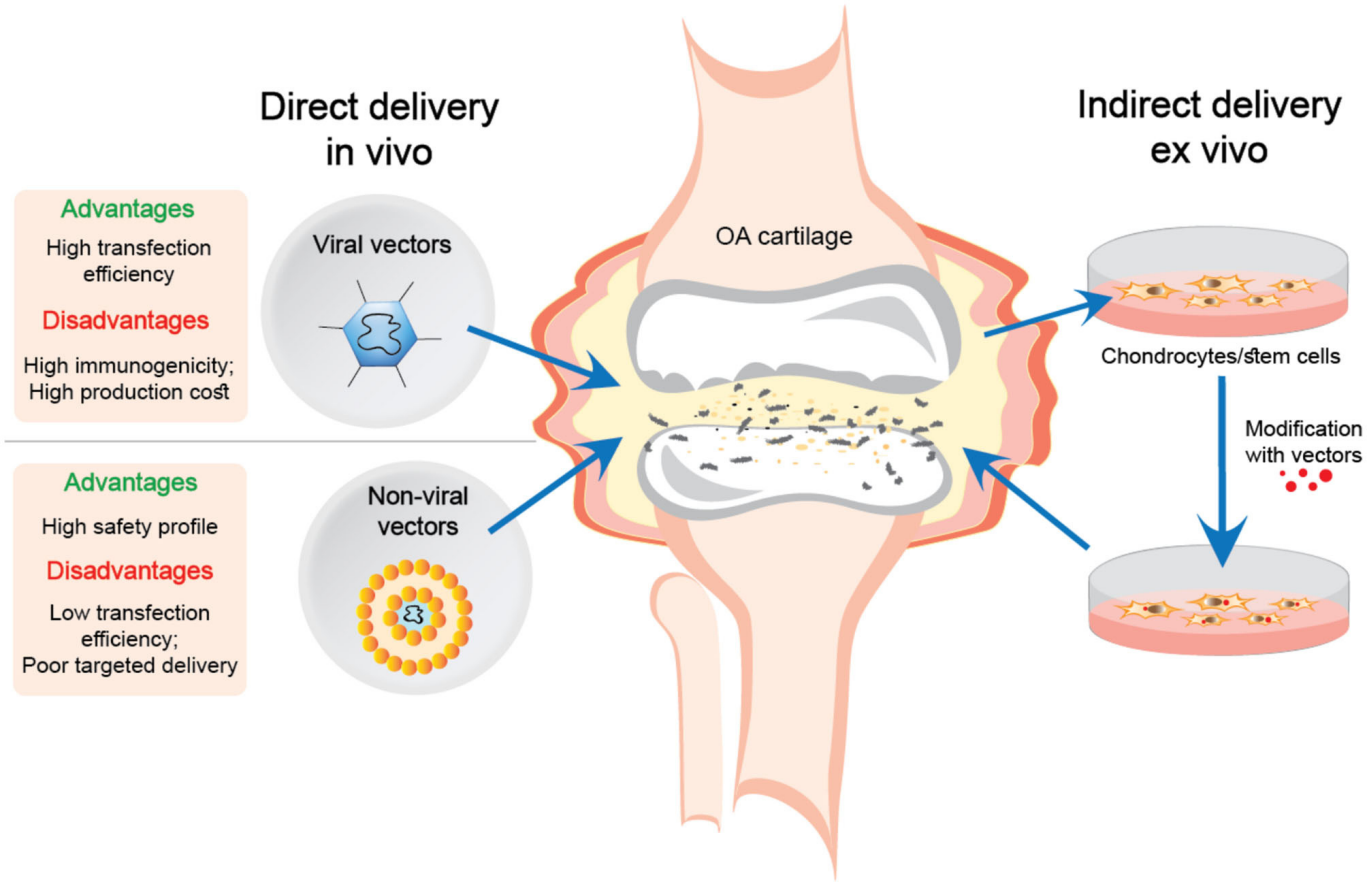
Direct and indirect delivery of viral and non-viral nucleic acid transfer systems into OA joints.

There are two main approaches for gene delivery using viral and non-viral gene transfer systems for treating articular cartilage defects in OA, or following joint trauma. The first is direct injection into the joint cavity *in vivo*, while the second involves the delivery of *ex vivo* manipulated cells (see Figure 1).

The *ex vivo* delivery of genes offers more advantages compared to direct injection of gene delivery systems. Extracting cells from the tissue and transfecting the genes *in vitro* is more efficient than introducing vectors directly into a living organism. Moreover, this procedure is much safer for delivering the genes in viral vectors, which eliminates the direct contact of the virus with the body (Evans et al., 2018).

Regenerative strategies for OA have traditionally incorporated cell-based approaches, including both native primary chondrocytes as well as stem cells such as mesenchymal stem cells (MSCs) or medicinal signaling cells, as proposed by Arnold Caplan (Richardson et al., 2010; Caplan, 2017, 2019) and pluripotent stem cells (PSCs) (Sakata et al., 2015; Murphy et al., 2018). Autologous chondrocyte implantation (ACI) was demonstrated as one of the most effective tools for cartilage restoration and even suggested to be used as first-line treatment in certain patients (Welch et al., 2016). However, a detailed discussion of recent progress in this area is way beyond the scope of this article and we refer the readers to some of recent publications (Fellows et al., 2016; Kobolak et al., 2016; Kalamegam et al., 2018). Nevertheless, stem cell therapies, including MSCs derived from various tissue sources, are considered potential to treat focal cartilage lesions due to their ability to differentiate into chondrogenic lineage (Madry et al., 2011; Uzieliene et al., 2018). However, the majority of cartilage repair studies using MSCs have failed or produced disappointing results so far due to a number of complicating factors, including insufficient capacity for chondrogenic differentiation, poor potential for immunomodulation or the development of hypertrophy (Mueller and Tuan, 2008; Mueller et al., 2010; Somoza et al., 2014). Therefore, viral and non-viral approaches may be used to enhance chondrogenic differentiation of MSCs by stimulating it with genes encoding growth factors or transcription factors required for chondrogenic response in MSCs (Leijten et al., 2014; Raisin et al., 2016). Moreover, gene therapies can enhance MSC immunomodulatory properties as well as stimulate anabolic chondrocyte responses in damaged cartilage or even inhibit anti-chondrogenic factors (Lolli et al., 2018).

Genetically engineering MSCs that can attach to the ECM in cartilage was a concept that was tried and tested more than a decade ago (Robbins et al., 2003) but now the emphasis appears to have shifted to modulating chondrocyte metabolism (Pirozzi et al., 2018), paracrine activity (Madry et al., 2004) and altering the inflammatory milieu of the microenvironment (Matta et al., 2015).

### Viral Gene Transfer Systems for OA

An example of cell therapy using genetically engineered cells is TissueGene C, a biological drug incorporating both cell and gene therapy. This therapy consists of a mixture of irradiated allogeneic primary chondrocytes and GP2-293 cells over-expressing transforming growth factor β1 (TGF-β1), whose gene was transduced using a retroviral vector into this protein packaging cell line (Lim et al., 2017). The GP2-293 cells are irradiated using gamma rays or x-rays to render them replication incompetent. This mixture is then injected into the OA knee joint and this has been shown to significantly promote cartilage regeneration in rabbits (Lee et al., 2001; Song et al., 2005). This approach has been demonstrated to be an effective and safe procedure in Phase 1 and Phase 2 clinical studies and was approved for treating patients with moderate knee OA in South Korea (Evans et al., 2018; Grol and Lee, 2018). In the United States TissueGene-C has recently entered Phase 3 clinical trials (Mobasheri, 2020).

The recent development of the CRISPR/Cas9 technology, and the award of the Nobel Prize in Chemistry to the inventors has opened up an exciting new avenue for easy and efficient gene editing in many diseases, including OA. CRISPR-based gene editing seems to be feasible for the development of new therapeutic strategies for OA treatment. Adeno-associated virus, which expressed CRISPR/Cas9 components to target each of the genes encoding matrix metalloproteinase 13 (MMP13), interleukin 1β (IL-1β), and nerve growth factor (NGF), was intra-articularly injected in a surgically induced OA in mice (Zhao et al., 2019). The results of this study suggest that multiple ablation of these genes provide benefits for both pain management and joint structure maintenance.

Adeno-associated viral vectors have been clinically adapted as an efficient tool for gene therapy in different osteoarticular disorders, as described by Cucchiarini and co-workers (Cucchiarini et al., 2015). Studies in rat MSCs have shown that transfection with adenoviral and lentiviral vectors can achieve efficiencies of up to 70 and 95%, respectively compared to only 25% with the non-viral liposome-based vector Lipofectin (McMahon et al., 2006). However, despite all the advantages viral gene delivery can propose, these systems have been shown to induce an inflammatory response in joints, which can cause different side effects (Evans et al., 2006; Saraf and Mikos, 2006; Bellavia et al., 2018). Moreover, the development of viral vectors is more expensive than non-viral, and their direct injection might potentially spread viruses to other organs, which is an undesirable consequence of using such vectors. The non-viral gene delivery systems are easy to handle, easy to synthesize, they have low immunogenicity and less expensive, as compared to viral gene transfection (Saraf and Mikos, 2006; Graceffa et al., 2018).

Although viral gene delivery strategy application is more widely used due to its efficiency, significant effort has gone into enhancing the transfection efficiency of non-viral delivery, making them promising tools for further application in clinical studies.

### Non-viral Gene Transfer for Cartilage Repair in OA

**Figure 2:**
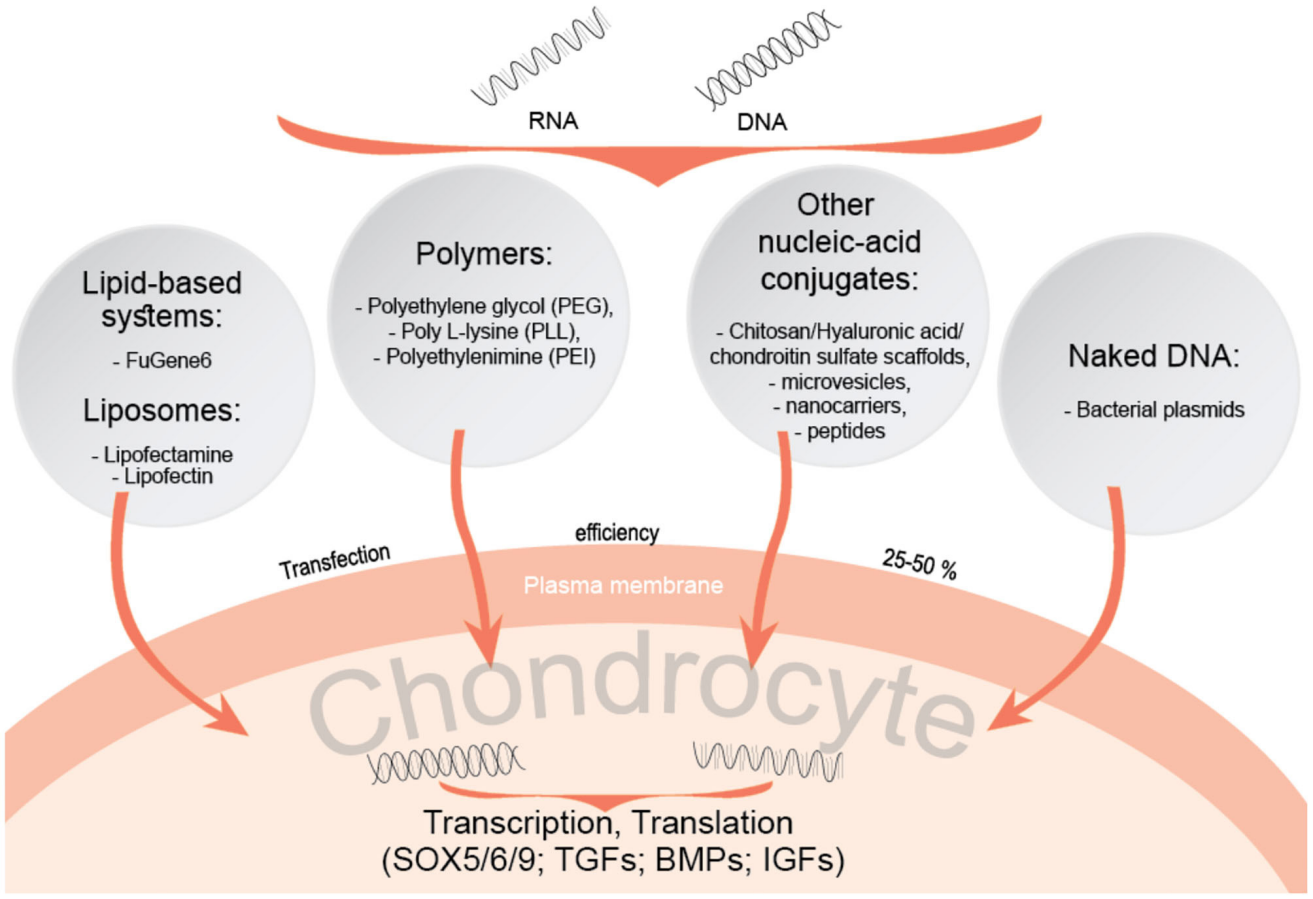
Non-viral nucleic acid-conjugating systems for gene delivery into chondrocytes.

Different non-viral gene transfer methods have been proposed in order to induce chondrogenesis in MSCs (Raisin et al., 2016). Scaffold systems, composed of natural components related to cartilage tissue, are tested for enhancing chondrogenesis of MSCs with gene delivering agents. Transcription factors such as SOX-5, 6 and 9, and growth factors (GFs), including transforming growth factors (TGFs) and bone morphogenetic proteins (BMPs) have already been investigated in this context (Figure 2). In studies of adipose tissue-derived MSCs *in vivo* aiming to evaluate the efficacy of TGF-β_2_ and BMP-7 in osteochondral defects, these growth factors were immobilised in scaffolds to overcome limitations associated with their structural instability and short half-lives (Im and Lee, 2010). Such scaffolds can also be used for transcription factor gene delivery, as demonstrated by enhanced chondrogenesis of adipose stem cells (ASCs) on a porous polylactide-co-glycolide (PGLA) scaffold, containing plasmid DNA encoding the SOX trio genes (SOX5, SOX6, and SOX9) and slowly releasing them to transfect ASCs seeded in the scaffold (Im et al., 2011). Therefore, chondrogenic transcription factors and growth factors can also be incorporated into non-viral gene therapy strategies for future therapeutic development. A study based on previously mentioned study, applying an improved and more advanced method by impregnating a porous poly-lactide-co-glycolide (PGLA) scaffold with SOX5, SOX6, and SOX9, resulted in enhanced chondrogenesis of adipose stem cells (Im et al., 2011). PLGA-based scaffold study including the same combination of SOX5/6/9 transcription factors but instead of adipose stem cells, hMSCs were obtained from bone marrow (Park et al., 2011). A combination of SOX5/6/9 genes were transfected into hMSCs, which resulted in compacted and highly distributed gene-transfected hMSC pellets, strongly stained by antibodies against type II collagen and aggrecan, suggesting this approach to be a suitable method for non-viral gene delivery. Another study on targeted delivery of SOX5, 6, and 9 into cells, but using different scaffold model, the porous collagen-based scaffold in combination with hyaluronic acid (CHyA), demonstrated chondrogenic differentiation of MSCs into phenotypically stable chondrocytes, kept all of the articular cartilage-like ECM following implantation *in vivo*, and inhibiting endochondral ossification (Raftery et al., 2020). TGF-β and SOX9 have been proposed to be the most potent chondrogenic factors for co-delivery into bone marrow-derived MSCs. Although SOX9 transcription factor alone does not induce chondrogenesis, a technique including a combination with heparinized TGF-β3-modified and PLGA scaffolds facilitated the simultaneous delivery of both genes and prevented dedifferentiation of transfected hMSCs.

Polymer based systems, including poly L-lysine (PLL), polyethylenimine (PEI) or polyethylene glycol (PEG) are also of great interest for gene delivery into cells. These polymers form strong electrostatic complexes with nucleic acids, which facilitates their entry into cells (Raisin et al., 2016; Song and Park, 2020). Polymer complexes with hyaluronic acid (HA) enhance more efficient gene delivery, as HA binds to the MSC CD44 receptor, enabling complexes to enter cells. Such methods were used for delivering SOX genes into MSCs, strongly stimulating their chondrogenic differentiation (Song and Park, 2020). Similar gene carriers using chitosan-graft-PEI (CP)/DNA nanoparticles (Lu et al., 2014), as well as HA-chitosan modified systems (Lu et al., 2011) have been proposed as an efficient way for delivering the genes to chondrocytes and synoviocytes.

Various materials for delivery and their physiochemical properties also play an important role in order to ensure the efficient targeting of cells and tissues, for example, biodegradable lipid and polymeric nanoparticles exhibit an important advantage over inorganic nanoparticles (Wells, 2010; Nayerossadat et al., 2012; Mashel et al., 2020). As previously mentioned, non-viral gene transfer methods include different kinds of liposomes or other nucleic acid conjugates and are applied for both local delivery and *ex vivo* extracted cells, even though their transfection efficiency is much lower compared to viral gene transfer (Robbins et al., 2003). However, non-viral gene transfection also proposes promising results. For instance, the lipid based non-viral gene transfection reagent “FuGene 6” has been used in C28/I2 chondrocytes and shown to produce reliable results but with a transfection efficiency of only 30% and cell viability of more than 95% (Greco et al., 2011). In this study, C28/I2 cells were transfected with the gene encoding BMP-2, which resulted in a significant increase in type II collagen and aggrecan gene expression.

The FuGene 6 transfection reagent was part of the early preclinical development strategy for expressing the human IL-10 transgene in HEK-293 cells (Watkins et al., 2020), which led to the formulation of a naked DNA plasmid in a vehicle consisting of PBS and D-mannose and subsequent testing in a translational model of OA in beagle dogs. The therapy was well-tolerated during a period of 6 months with a significant decrease in pain, according to the behavior of the animals. This approach has been proposed as the scientific basis for future clinical trials in humans.

Liposomes are one of the major nucleic acid carrying systems proposed as potential tools to replace viral-vectors. These cationic lipids consist of positively and negatively charged groups and have an affinity to combine negatively charged DNA. They form bilayered vesicles and serve as DNA transporting vehicles (Clanchy and Williams, 2008). However, transfection efficiency of these systems is weaker than the lipid-based systems, for instance, commercialized product Lipofectin has been shown to be less effective in gene transfer as compared to FuGene 6 (Stöve et al., 2002; Graceffa et al., 2018). In addition to lipids, other nanocarriers, such as nanomicelles, nano-microspheres were studied (Chen et al., 2018). The mix of chitosan, HA and chondroitin sulfate (CS) was proposed as an efficient nano-microsphere for GDF-5 plasmid transfer into the articular cavities of rabbits with developed OA and demonstrated low cytotoxicity on chondrocytes *in vitro* and also a high transfection efficiency *in vivo* (more than 60%), promoting ECM production *in vivo*, as compared to control group (Chen et al., 2018).

Another potential option for targeted gene delivery includes DNA-carrying peptides. Chondrocyte-affinity peptide (CAP), which interacts specifically with chondrocytes was covalently modified to bind to CAP forming a non-viral vector (Pi et al., 2011). These constructs were injected into rabbit knee joints and were shown to be specifically taken up by chondrocytes, as compared to single PEI vectors (Pi et al., 2011). In addition to DNA conjugating systems, naked-DNA can be used as a vector for gene delivery to target tissues, even though this method is relatively expensive and requires frequent administration, which means that this approach is mainly experimental. Such vectors have the potential to be used in the treatment of OA. Naked-DNA method involves additional physical techniques (hydrodynamic, ultrasound, electroporation) in order to transfer genes to cells, which highlights safety issues associated with this non-viral gene therapy compared to DNA carrying systems (Clanchy and Williams, 2008).

Another potential disease-modifying strategy applied for OA is using messenger RNA (mRNA)-conjugating systems as a disease-modifying strategy for OA. Nano-micelles of PEG carrying the cartilage anabolic factor runx-related transcription factor-1 (RUNX1) mRNA have been shown to significantly suppress the progression of OA after intra-articular injection in a mouse OA model (Aini et al., 2016).

Therefore, significant progress has been made in the field of non-viral gene transfer, increasing the efficiency of the tools and available methods, which might lead to new perspectives and therapeutic strategies for future clinical studies.

## Future Trends and Perspectives

Future non-viral gene therapy technologies have a great potential to transform development of new therapeutics for OA, even though they have two critical weaknesses, including transient gene expression and low transfection efficiency, as compared to viral gene therapy (Li and Huang, 2000; Ramamoorth and Narvekar, 2015). On the other side, viral gene therapy includes the risk of activating the innate immune system and causing local inflammation. Therefore, non-viral gene transfer seems to be a more suitable and potentially safer option particularly due to the fact that OA is a disease characterized by low-grade inflammation (Mobasheri et al., 2019b). We have introduced the main strategies of applying both gene transferring systems *in vivo* and *ex vivo*, where an *ex vivo* method offers more advantages. As discussed in this mini review, two successful examples of *ex vivo* therapy, TissueGene C (Lim et al., 2017) and FuGene 6 drugs (Greco et al., 2011) have been even clinically approved. Moreover, improving stem cell chondrogenic differentiation with different stimulating factor genes is also one of the main strategies of *ex vivo* gene therapies.

The concept of introducing gene-based therapeutic approaches for the treatment of OA requires safe and targeted strategies. Non-viral gene therapies include lipid-based systems, polymers, natural components, or even naked plasmids, as safe ways of introducing genes into cells. These methods allow penetration of gene delivery vectors to the articular cartilage ECM and reaching the chondrocytes to modify them genetically (Li and Huang, 2000; Lu et al., 2014). Future studies should focus on enhancing the efficacy of vector delivery. To ameliorate vector penetrability and gene delivery to the target cells through abundant and complex ECM of articular cartilage, different delivery method enhancements, like electroporation, microbubble ultrasound, optoporation, magnetofection should be investigated and compared (Mashel et al., 2020). However, these techniques are beyond the scope of this mini-review for a detailed discussion. Furthermore, among various materials used for gene delivery, biodegradable structures as lipids and polymers exhibit greater adaptability and use in *in vivo*, which is one of the key factors for their broad prospects in further analysis. These systems are currently under investigation in preclinical studies with the ambition to progress to future clinical applications (Mashel et al., 2020). In conclusion, progress has been made in the development of methods and technologies to deliver gene therapy and test them in experimental and translational models of OA. However, additional studies are needed to optimize the transfection efficiency and duration of gene expression.

## Author Contributions

IU, UK, EB, and AM: conceptualization, review methodology and acquisition of resources, writing – revision, and review and further editing. IU: writing – original draft. EB and AM: supervision. AM: final submission and funding acquisition. All authors contributed to the article and approved the submitted version.

## Conflict of Interest

AM is a member of the Scientific Advisory Board of Kolon TissueGene (Rockville, MD) and has received research funding from Kolon TissueGene and Merck KGaA, Darmstadt, Germany. The remaining authors declare that the research was conducted in the absence of any commercial or financial relationships that could be construed as a potential conflict of interest.

## Abbreviations

BMPs: bone morphogenetic proteins
CAP: chondrocyte-affinity peptide
CRISPR/Cas9: clustered regularly interspaced palindromic repeats-associated nuclease 9
CS: chondroitin sulfate
DMOADs: disease-modifying osteoarthritis drugs
GFs: growth factors
HA: hyaluronic acid
IL-1β, 10: interleukin 1β, 10
MMP13: matrix metalloproteinase 13
MSCs: mesenchymal stem cells
NGF: nerve growth factor
NSAIDs: non-steroidal anti-inflammatory drugs
OA: osteoarthritis
OARSI: Osteoarthritis Research Society International
PEG: polyethylene glycol
PEI: polyethylenimine
PLL: poly L-lysine
PSCs: pluripotent stem cells
RUNX: runt-related transcription factor
SOX-5, SOX-6, SOX-9: transcription factor SOX 5, 6, 9
TGF-β1: transforming growth factor β1.
